# Metagenomic-based pathogen surveillance for children with severe pneumonia in pediatric intensive care unit

**DOI:** 10.3389/fpubh.2023.1177069

**Published:** 2023-06-15

**Authors:** Meijin Li, Jing Wang, Zhongwei Yao, Hailing Liao, Shufen Su, Xuying Yang, Mingzhou Xie, Yinan Zheng

**Affiliations:** ^1^Department of Pediatric Intensive Care Unit, Guangdong Women and Children Hospital, Guangzhou, China; ^2^Department of Scientific Affairs, Hugobiotech Co., Ltd., Beijing, China

**Keywords:** severe pneumonia, PICU, mNGS, pathogens, co-infection

## Abstract

**Background:**

Pneumonia is a significant cause of morbidity and mortality in children. Metagenomic next-generation sequencing (mNGS) has the potential to assess the landscape of pathogens responsible for severe pulmonary infection.

**Methods:**

Bronchoalveolar lavage fluid (BALF) samples of 262 children with suspected pulmonary infections were collected from April 2019 to October 2021 in the Pediatric Intensive Care Unit (PICU) of Guangdong Women and Children Hospital. Both mNGS and conventional tests were utilized for pathogen detection.

**Results:**

A total of 80 underlying pathogens were identified using both mNGS and conventional tests. Respiratory syncytial virus (RSV), Staphylococcus aureus and rhinovirus were the most frequently detected pathogens in this cohort. The incidence rate of co-infection was high (58.96%, 148/251), with bacterial-viral agents most co-detected. RSV was the main pathogen in children younger than 6 months of age, and was also commonly found in older pediatric patients. Rhinovirus was prevalent in children older than 6 months. Adenovirus and Mycoplasma pneumoniae were more prevalent in children older than 3 years than in other age groups. Pneumocystis jirovecii was detected in nearly 15% of children younger than 6 months. Besides, influenza virus and adenovirus were rarely found in 2020 and 2021.

**Conclusions:**

Our study highlights the importance of using advanced diagnostic techniques like mNGS to improve our understanding of the microbial epidemiology of severe pneumonia in pediatric patients.

## Introduction

Pneumonia or lower respiratory tract infection causes considerable morbidity and mortality in children worldwide, accounting for nearly 12% of deaths in Pediatric Intensive Care Units (PICUs) (1, 2). The pathogens responsible for pulmonary infection in hospitalized children have been evaluated by several studies (3–5). Recently, a landmark study in children with severe lower respiratory tract infection (LTRI) showed that the most common causative pathogens were respiratory syncytial virus, *Haemophilus influenzae* and *Moraxella catarrhalis*, and also highlighted the occurrence of viral-bacterial co-infection (6). This study “advances the understanding of LRTI microbial epidemiology,” but there are still few research focus on the etiology of children with severe pulmonary infection.

Children with severe pneumonia face a high risk of adverse outcomes. Accurate diagnosis based on pathogen detection is crucial for patient care. Traditional culture-based detection methods often fail to identify uncultivatable or fastidious pathogens and are susceptible to empirical antibiotic treatment (7). PCR assays can be a useful tool for detecting pathogens that are difficult or impossible to culture. However, these assays require prior knowledge of the specific pathogen being tested for, which can limit their utility in cases where the causative agent is completely unknown. Emerging as a rapid and untargeted technique, metagenomic next generation sequencing (mNGS) has the potential to investigate the whole landscape of pathogens in samples (8, 9). As mNGS is increasingly used in infectious disease diagnosis, it has affected clinical therapeutic strategies by identifying pathogens missed by traditional tests (10, 11). The utility of mNGS in pathogen detection for complex respiratory samples is still in the early stage, but it holds promise to provide etiological and epidemiological information for understudied patient populations, which has implications for early prevention and treatment (6).

In this study, we collected bronchoalveolar lavage fluid (BALF) samples from children with severe pneumonia who admitted to a PICU for mNGS and conventional tests. The characteristics of pathogen profiles were assessed, and the spectrum of pathogens in patients with different ages were analyzed. We also mentioned the differences in respiratory virus detection before and after the start of coronavirus disease 2019 (COVID-19) pandemic.

## Methods

### Study design

Patients with suspected pulmonary infections who admitted to the Pediatric Intensive Care Unit (PICU) of Guangdong Women and Children Hospital from April 2019 to October 2021 were enrolled. The diagnosis of pulmonary infection was based on (1) chest X-ray or computed tomography revealed new-onset patchy infiltrating shadows, leaf or segmental consolidation shadows, ground glass shadows, or interstitial changes, with or without pleural effusion and (2) at least one of the following typical clinical characteristics: (a) new-onset cough, sputum production, dyspnoea, chest pain, or exacerbation of existing respiratory symptoms, (b) fever, (c) clinical signs of lung consolidation or moist rales, and (d) peripheral leukocytosis (>10 × 10^9^/L) or leucopenia (<4 × 10^9^/L), with or without left shift of cell nucleus. BALF samples from the patients enrolled were collected, and tested for conventional methods and mNGS of DNA and RNA. Conventional methods included culture, antibody measurement (IFA: EUROIMMUN, including 8 pathogens: respiratory syncytial virus, adenovirus, Influenza A, Influenza B, parainfluenza, *Mycoplasma pneumonia*, *Chlamydia pneumonia*, *Legionella pneumophila*) and PCR (including 9 pathogens: cytomegalovirus, Epstein–Barr virus, *Mycoplasma pneumonia*, *Mycobacterium tuberculosis*, enterovirus, respiratory syncytial virus: Da’an Gene Co., Ltd., Guangzhou, China; rhinovirus, bocavirus: Land Medical Co., Ltd., Hubei, China; adenovirus:Sansure Biotech Co., Ltd., Hunan, China). The final clinical diagnoses were based on the results of mNGS, conventional methods, and professional judgement and treatment by clinicians.

The study protocol was approved by the Clinical Research Ethics Committee of Guangdong Women and Children Hospital. Parents or guardians of participants provided written informed consent.

### Specimen processing and sequencing

According to manufacturer’s instruction of QIAamp DNA Micro Kit (QIAGEN, Hilden, Germany), DNA was extracted and purified. We used QIAamp Viral RNA Mini Kit (QIAGEN, Hilden, Germany) to extract RNA, which was further transcribed in reverse using SMART MMLV Reverse Transcriptase kit (Takara Biotechnology Co. Ltd., Dalian, China). The concentration and quality of extraction were tested through Qubit 4.0 (Thermo Fisher Scientific, MA, United States). DNA libraries and RNA libraries were constructed using QIAseq Ultralow Input Library Kit (QIAGEN, Hilden, Germany) and TruePrep DNA Library Prep Kit (Vazyme, Jiangsu, China), respectively. The inspected libraries were sequenced on Nextseq 550 platform (Illumina, San Diego, United States).

### Bioinformatics analysis

The obtained sequencing raw data were filtered to remove adapter and low-quality, low-complexity, and shorter reads (<35 bp). Human reads were removed by mapping to human reference genome (hg38) using bowtie2 to obtain clean reads. Subsequently, using Burrows-Wheeler Alignment (12), the obtained clean sequences were aligned with microbial Pan-genome database, which was conducted according to Reference Sequence Database of National Center for Biotechnology Information^1^ (7, 13).

In parallel with the samples, negative and positive controls were also set for mNGS detection with the same procedure and bioinformatics analysis. The specific reads number and reads per million (RPM) of each detected pathogen were calculated. For the detected bacteria and fungi, a positive mNGS result was defined when the microorganism was not detected in the negative control (“No template” control, NTC) and genome coverage of detected sequences belonged to this microorganism ranked top10 of the same kind of microbes or when its ratio of RPM_sample_ to RPM_NTC_ was (RPM_sample_/RPM_NTC_) > 10 if the RPM_NTC_ ≠ 0. For viruses, a positive mNGS result was considered when it was not detected in NTC and at least 1 specific read was mapped to species or when RPM_sample_/RPM_NTC_ was >5 if the RPM_NTC_ ≠ 0.

### Statistical analysis

Counts and percentages were presented for independent binomial variables. Interquartile ranges (IQRs) were calculated using IBM SPSS 25.0. Chi-square test was performed to compare the frequencies, and *p* value <0.05 were considered statistically significant. The data were analyzed using R 4.1.1.

## Results

### Clinical characteristics

A total of 262 pediatric patients with suspected pulmonary infectious diseases were enrolled in our study, including 167 males and 95 females. The age of these patients ranged from 8 days to 11 years old, with a median age of 5.5 months (IQR:2.875–13 months) (Table 1). The main clinical symptoms included fever (*n* = 98), apnea or respiratory distress (*n* = 53), dystonia (*n* = 51), gastrointestinal dysfunction (*n* = 116), cough (*n* = 189), gurgling with sputum (*n* = 50), dyspnea (*n* = 39), wheezing (*n* = 43) and moist crackles (*n* = 162) (Table 1). BALF samples were collected from these enrolled patients for both mNGS and conventional tests.

**Table 1:** Physical information and clinical characteristics of the enrolled pediatric patients.

	Count	Percentage
*Sex*		
Male	167	63.74%
Female	95	36.26%
*Age*		
< 6 months	142	54.20%
6 month – 1 years	37	14.12%
1 years – 3 years	49	18.70%
>3 years	34	12.98%
*Clinical characteristic*		
fever	116	44.27%
cough	189	72.14%
tachypnea	144	54.96%
gurgling with sputum	50	19.08%
dyspnea	39	14.89%
wheezing	43	16.41%
moist crackles	162	61.83%
*Immunity status*		
Compromised	6	2.29%
Normal	173	66.03%
Unclear	83	31.68%
*Type of infection*		
Community-acquired pneumonia (CAP)	220	83.97%
Ventilator-associated pneumonia (VAP)	31	11.83%
**Comorbidities**	89	35.46%

Based on the results of mNGS and conventional tests as well as therapeutic outcomes, 251 children were diagnosed with severe pneumonia, including 220 with community-acquired pneumonia (CAP) and 31 with ventilator-associated pneumonia (VAP). Of these children, 89 had comorbidities, including congenital heart disease (*n* = 38), bronchopulmonary dysplasia (*n* = 33), esophageal atresia/esophagotracheal fistula (*n* = 13), gastroesophageal reflux (*n* = 13), pulmonary sequestration (*n* = 3), spinal muscular atrophy (*n* = 3), pulmonary cystadenoma (*n* = 1), diaphragmatic hernia (*n* = 1) and diaphragmatic eventeration (*n* = 1).

### Pathogen profiles

**Figure 1:**
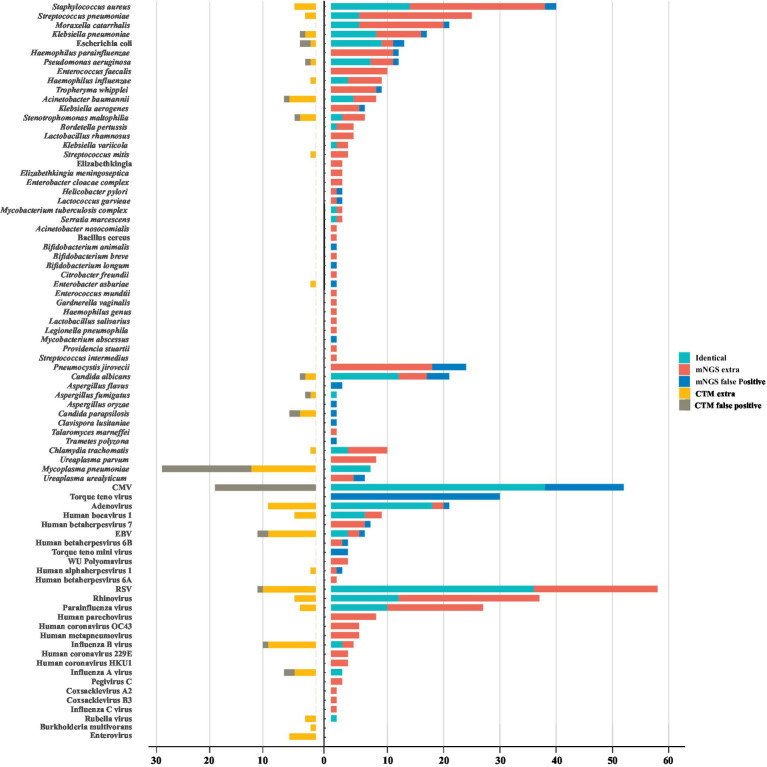
The landscape of potential pathogens detected by metagenomic next generation sequencing (mNGS) and conventional methods. The pathogens consisted of identical detection (the green) of mNGS with conventional tests (CTM), extra detection (the red) of mNGS, false positive detection (the blue) of mNGS, extra detection (the yellow) of CTM, false positive detection (the grey) of CTM. The false positive detection of mNGS represent those microorganisms detected only by mNGS and diagnosed as non-causitive pathogens. The false positive detection of CTM represent those microorganisms detected only by CTM and diagnosed as non-causitive pathogens. CMV, cytomegalovirus; EBV, Epstein–Barr virus; RSV, respiratory syncytial virus.

A total of 80 underlying pathogens were identified in children with severe pneumonia, including bacteria (50%, 40/80), fungi (11.25%, 9/80), DNA viruses (13.75%, 11/80), RNA viruses (20%, 16/80) and mycoplasmas/chlamydia (5%, 4/80; Figure 1). mNGS detected almost all pathogens (78/80), with a detection rate of 97.61% (245/251), which is much higher than that of conventional methods (73.31%, 184/251).

The most frequently detected bacteria in this cohort were *Staphylococcus aureus* (17.53%, 44/251), *Streptococcus pneumoniae* (10.76%, 27/251) and *M. catarrhalis* (8.37%, 21/251). There were 24 bacteria that conventional methods failed to detect, and among them, the pathogenicity of 20 bacteria could not be ruled out, including *Haemophilus parainfluenzae*, *Enterococcus faecalis*, *Tropheryma whipplei*. The dominant fungi identified in pulmonary infection were *Pneumocystis jirovecii* (9.56%, 24/251) and *Candida albicans* (9.16%, 23/251). *P. jirovecii* were only detected by mNGS, of which 25% (6/24) of them were false positives, and others were detected in immunocompromised children or those with long-term medication for underlying diseases, including one case of VAP. Though rarely pathogenic, cytomegalovirus (CMV) were found in 28.69% (72/251) of the pediatric patients. Among these cases, 52.78% (38/72) were identified by both mNGS and conventional methods, and most of them (25/38) being found in immunocompetent children. Adenovirus (11.95%, 30/251) was the most identified causative DNA virus. For RNA viruses, respiratory syncytial virus (RSV) contributed most (27.49%, 69/251) to detection, followed by rhinovirus (16.33%, 41/251) and parainfluenza virus (11.95%, 30/251). The majority of the detected RNA viruses (60%, 9/15) were not identified by conventional methods (Figure 1). In addition, *Mycoplasma pneumoniae* were identified in 36 children by antibody measurements, but 17 of them were diagnosed as false positive cases according to mNGS negative results (Figure 1).

### High incidence of co-infection

According to final clinical diagnosis, mixed infections caused by different types of pathogens (bacteria, fungi, viruses, mycoplasmas, chlamydia) were identified in 148 (58.96%) children of this cohort, including bacterial-viral co-infection (45.02%, 113/251), bacterial-fungal co-infection (12.75%, 32/251), fungal-viral co-infection (12.75%, 32/251), bacterial-other co-infection (5.18%, 13/251), fungal-other co-infection (1.99%, 5/251) and viral-other co-infection (8.37%, 21/251; Table 2). Bacterial-viral agents were detected simultaneously in 98 cases using mNGS, with *S. pneumonia*-RSV (*n* = 9) most co-detected.

**Table 2:** The types of infections of enrolled patients based on clinical diagnosis.

	Count	Percentage
bacterial-fungal co-infection	7	2.67%
bacterial-viral co-infection	84	32.06%
fungal-viral co-infection	7	2.67%
bacterial-fungal-viral co-infection	22	8.40%
bacterial-fungal-viral-mycoplasmal co-infection	2	0.76%
bacterial-mycoplasmal co-infection	2	0.76%
bacterial-chlamydial co-infection	3	1.15%
bacterial-fungal-mycoplasmal co-infection	1	0.38%
bacterial-viral-mycoplasmal co-infection	5	1.91%
fungal-mycoplasmal co-infection	1	0.38%
fungal-viral-mycoplasmal co-infection	1	0.38%
viral-mycoplasmal co-infection	9	3.44%
viral-chlamydial co-infection	4	1.53%
bacterial infection	47	17.94%
viral infection	42	16.03%
fungal infection	4	1.53%
mycoplasmal infection	7	2.67%
chlamydial infection	3	1.15%
non-infection	11	4.20%

As the most common pathogenic RNA viruses in this study, RSV was detected with bacterial agents in 35 (50.73%) children, of which all were diagnosed with bacterial-viral co-infection. Beside *S. pneumonia*, the most identified bacteria with RSV was *S. aureus* (*n* = 7). *H. influenzae*-RSV co-infection was identified in 3 cases (Supplementary Figure S1). For children infected with adenovirus, 23.81 and 47.62% of them were co-detected with bacterial and other viral agents, respectively (Supplementary Table S1). Not all of the microbes listed here were etiological pathogens, but we proposed that the threat to patients from coexistence of non-causative microbes (except for contaminants) or potential pathogens with etiological pathogens should not be overlooked. The co-detection of *M. pneumoniae* by mNGS was shown in Supplementary Table S2.

### Pathogens identified in different age group

**Figure 2:**
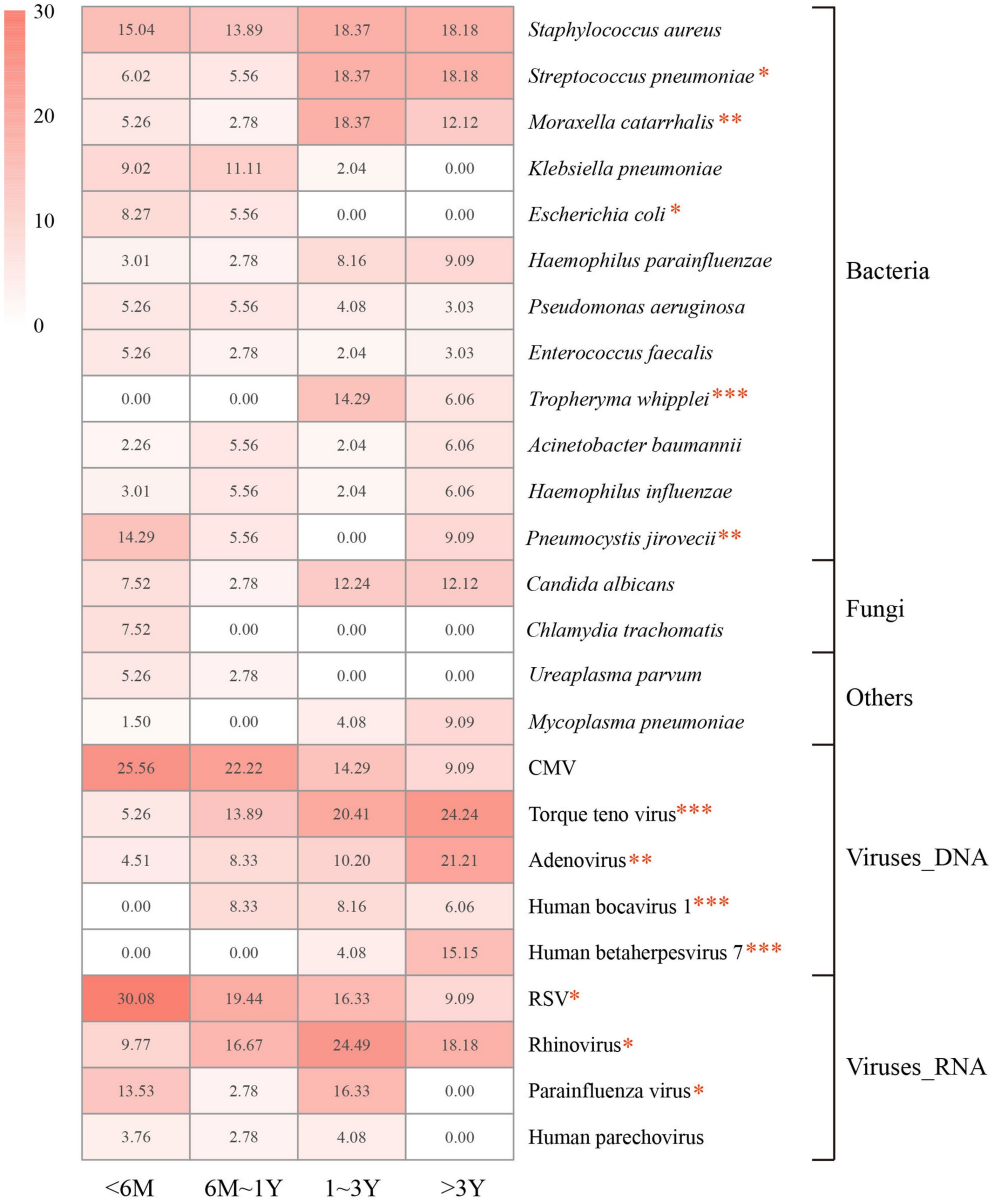
Proportions of the dominant pathogens detected in each age group. Proportions are calculated as the case numbers of the pathogen out of the total number of patients in the age group. M, months of age; Y, years of age. Statistical significance was determined by *χ*^2^ test. *p < 0.05. **p < 0.01. ***p < 0.001. CMV, cytomegalovirus; RSV, respiratory syncytial virus.

The spectrums of detected pathogens were further compared in different age groups. Twelve potential pathogens were identified statistically different across age groups in children with pulmonary infection, including *S. pneumoniae* (*p* = 0.011), *M. catarrhalis* (*p* = 0.006), *Escherichia coli* (*p* = 0.038), *T. whipplei* (*p* < 0.001), *P. jirovecii* (*p* = 0.002), Torque teno virus (*p* < 0.001), Human bocavirus (*p* < 0.001), Human betaherpesvirus 7 (*p* < 0.001), adenovirus (*p* = 0.001), RSV (*p* = 0.014), rhinovirus (*p* = 0.011), and parainfluenza virus (*p* = 0.025; Figure 2).

The most frequently detected potential pathogens in children younger than 6 months of age were RSV (30.08%), CMV (25.26%), *S. aureus* (15.04%), and *P. jirovecii* (14.29%). The detection of RSV and *P. jirovecii* markedly decreased in other groups, while RSV (19.44%) was still the dominant pathogen detected in children aged 6 months to 3 years. Rhinovirus (24.49%) was the most identified pathogen in children aged 1–3 years, and was prevalent in children older than 6 months of age. The prevalence of adenovirus increased with age across all groups, and was most detected in children older than 3 years (21.21%).

### Differences in the detection of respiratory viruses during 2019–2021

**Figure 3:**
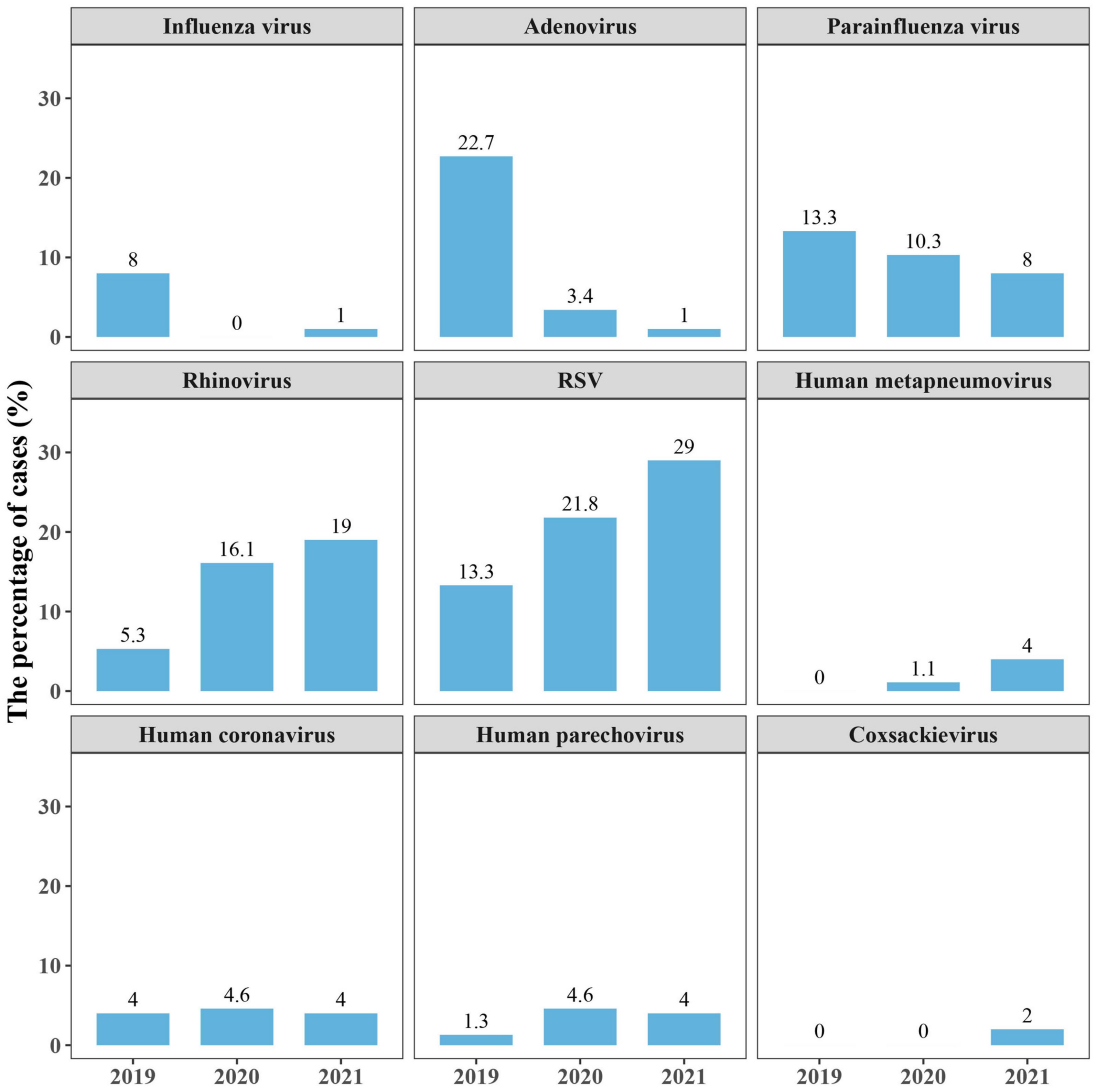
Proportions of several respiratory viruses detected in 2019–2021. Proportions are calculated as the case numbers of the pathogen out of the total number of patients in the year. RSV, respiratory syncytial virus.

We further analyzed the detection of respiratory viruses in different years to see if there were any differences before and after the start of the outbreak of coronavirus disease 2019 (COVID-19). The number of children admitted in 2019, 2020 and 2021 were 75, 87 and 100, respectively. The results showed that the activity of Influenza A/B/C virus, adenovirus markedly decreased in 2020, and kept low in 2021. Besides, the detection rate of parainfluenza virus was declining after 2019. There was no decline in the detection of rhinovirus and RSV during the pandemic period. The other respiratory viruses, such as Human metapneumovirus, Human coronavirus (229E, HKU1, OC43), Human parechovirus and Coxsackievirus (A2, B3), were rarely detected during the 3 years (Figure 3). SARS-CoV-2 was not detected in this study because COVID-19 patients were admitted to the designated provincial COVID-19 treatment hospital.

## Discussion

In this study, mNGS was used for pathogen detection in children with severe pneumonia who admitted to a PICU. BALF samples were collected and 251 children were finally diagnosed with pulmonary infections. mNGS identified 78 underlying pathogens in children with severe pneumonia, showing a high detection rate of 97.61%. The dominant pathogens were RSV, *S. aureus* and rhinovirus. High incidence rate (58.96%) of co-infection was present, with bacterial-viral agents most co-detected. The detection rate of 12 pathogens showed differences in children of different ages, and the activity of respiratory viruses showed differences before and after the outbreak of COVID-19.

Viruses were most common detected in children with severe pneumonia. RSV was the main causative virus, followed by rhinovirus. These results are consistent with previous researches (4, 6, 14, 15). RSV causes massive disease burden in children, with peak rates of lower respiratory tract infection in winter (16, 17). It is reported that rhinovirus was also the leading cause of pneumonia in children (18, 19), but it can be frequently detected in patients who were asymptomatic (6, 20). The pathogenicity of rhinovirus is currently difficult to define. Some viruses were commonly detected as non-causative pathogens, such as CMV and Torque teno virus. However, CMV has a high risk of reactivation in ICU patients, being associated with severe morbidity and mortality (21, 22). We proposed that not only causative pathogens should be noticed, and the pathogenicity of microorganisms should be taken based on case status.

Co-infections were commonly found in children with severe pneumonia. In this study, bacterial-viral agents were co-detected in 98 patients. There is increasing evidence demonstrating bacterial-viral co-infection were associated with disease severity (23–26). Opportunistic pathogenic bacteria, such as *S. aureus*, *S. pneumoniae*, *H. influenzae* and *M. catarrhalis*, are usually co-detected with respiratory viruses (15, 25, 27, 28). Incidence of bacterial co-infection with RSV, which were not uncommon in PICU (15, 27), accounted for approximately 50% in our study. For adenovirus-infected children, nearly 30% of them were detected with bacterial agents. These results further underscore the clinical importance of bacterial-viral infection in children with severe pulmonary infection.

In addition to bacterial-viral co-infections, other mixed infections were also found in this cohort. Viral-viral co-infection status may affect viral loads and cause more severe symptoms (29–31). The incidence rate of adenovirus-viral co-infection in this study is also high. DNA and RNA viruses were co-detected in about 25% of the patients. Besides, there were a few cases of *M. pneumoniae* pneumonia, and most of them were co-infected by other pathogenic agents according to mNGS results. This is in line with the findings that children with severe *M. pneumoniae* pneumonia have a higher rate of co-infection (32, 33). The advantage of mNGS in detecting the whole pathogens may shed light on the true burden of diseases from mixed infections.

Although RSV and rhinovirus are the leading causes of pneumonia in children, there are some studies indicating that influenza viruses were important contributor as well (34–36). Influenza viruses were only detected in a few patients in this study, and nearly vanished in 2020 and 2021. This might be due to the outbreak of COVID-19 in 2019 and subsequent non-pharmaceutical interventions (e.g., mask use, physical distancing, and staying home), which leaded to a decrease in the prevalence of respiratory viruses (37, 38). The spread of adenovirus and parainfluenza virus, which can cause epidemics of respiratory tract infection (39, 40), also curtailed in PICU during COVID-19 pandemic.

Our study found differences in the prevalence of several pathogens among children in different age groups. As previous reported (6), RSV was the most identified pathogens in children younger than 6 months of age, and rhinovirus was prevalent in children older than 6 months. Nevertheless, RSV was also commonly detected in other age groups of this cohort. The detection rate of adenovirus and *M. pneumoniae* in children over 1 years old were much higher than those in younger groups. *P. jirovecii*, which has a mortality rate of 20% ~ 62.5% in children with Human immunodeficiency virus (HIV) infection and young age, was detected in nearly 15% of children younger than 6 months, and showed low activity in older groups. These findings suggest that there are some differences in the epidemiology of childhood pneumonia between developed and developing countries, and further studies are required.

*Ureaplasma urealyticum*, *Ureaplasma parvum* and *Chlamydia trachomatis* were only identified in children younger than 1 year of age. These microbes, which could not previously be identified by conventional diagnostic tests, were increasingly recognized as pathogens in neonates (41). *Ureaplasma* and *Mycoplasma* infections can be transmitted vertically from mother to child, and are proved to be involved in the causation of preterm birth in pregnant women, placental inflammation and neonatal respiratory disease (42–44). The potential of mNGS in detecting whole pathogens of infectious diseases can provide more effective reference for clinicians, which are conducive to accurate and timely diagnosis.

In this cohort, *E. faecalis* was identified by mNGS in 10 children with CAP, including 2 who were immunodeficient. A recent study revealed that lysophosphatidic acid produced by pathogenic *E. faecalis* in the intestine is a virulence factor that can cause pediatric pneumonia, inducing immune responses in the lungs and blood (45). Although *E. faecalis* has been reported in CAP patients (46, 47), it tend to affect immunocompromised individuals in community settings (48). Enterococci are typically associated with nosocomial infections (49, 50), and clinical isolates are frequently resistant to antimicrobials (48, 51). Most of the children identified with *E. faecalis* in this study were co-detected with other bacteria, and had previously been admitted to other hospitals and received antibiotics prior to being transferred to our PICU. While we do not believe that *E. faecalis* is the primary cause of CAP in these cases, it cannot be ruled out as a possible pathogen. Therefore, we suggest that the pathogenicity of *E. faecalis* found in children in PICU should be carefully considered, particularly in immunocompromised and hospitalized patients.

Our study demonstrates the potential of mNGS as a valuable tool for pathogen detection in children with severe pneumonia. The rapid speed and wide-range detection capabilities of mNGS compared to traditional methods make it an attractive option for routine diagnostics. While various pathogen detection kits are available for respiratory pathogen detection (52, 53), they are limited by pre-assumed pathogens, whereas mNGS can identify unknown or novel pathogens in a single test. Unlike PCR assays that cannot reflect the activity status of pathogens, mNGS is capable of simultaneously detecting DNA and RNA, thereby providing a more comprehensive understanding of the detected pathogens. Nonetheless, the high cost of mNGS is still a major obstacle to its widespread adoption in clinical settings (8, 54). Additionally, the accuracy of mNGS results can be compromised by false positives due to contamination or sequencing errors, presenting a particular challenge in respiratory samples where distinguishing between true pathogens and colonization can be difficult for clinicians (55). To address this issue, various criteria have been proposed to define causality and prioritize potential pathogens for further testing (7, 56, 57). Future research should focus on developing more specific algorithms to improve the accuracy of mNGS and maximize its clinical utility. Despite these challenges, mNGS has the potential to revolutionize clinical diagnostics and improve patient outcomes.

There are also some limitations in this study. First, it was a single-center study and had a relative small sample size. Second, children older than 5 years of age were rarely enrolled, which could not present the etiology of older children. Third, the antibiotic resistance of causative pathogens cannot be determined in this study.

## Conclusion

We assessed the landscape of potential pathogens for pediatric severe pneumonia in PICU. RSV and rhinovirus were the main pathogens responsible for pulmonary infections. The prevalence of main pathogens showed differences in different age group, and the activity of Influenza virus and adenovirus markedly decreased during the COVID-19 pandemic. Our findings highlighted the high incidence rate of co-infections in children with severe pneumonia, with bacterial-viral agents most frequently co-detected. mNGS pathogen detection could provide more effective reference for accurate etiological diagnosis, and our results could help enhance our understanding of the microbial epidemiology of severe pneumonia in children.

## Data availability statement

The datasets presented in this study can be found in online repositories. The names of the repository/repositories and accession number(s) can be found at: https://ngdc.cncb.ac.cn/?lang=zh, PRJCA012811.

## Ethics statement

The studies involving human participants were reviewed and approved by the Clinical Research Ethics Committee of Guangdong Women and Children Hospital. Written informed consent to participate in this study was provided by the participants’ legal guardian/next of kin.

## Author contributions

All authors listed have made a substantial, direct, and intellectual contribution to the work and approved it for publication.

## Conflict of interest

JW, XY, and MX were employed by Hugobiotech.

The remaining authors declare that the research was conducted in the absence of any commercial or financial relationships that could be construed as a potential conflict of interest.

## Publisher’s note

All claims expressed in this article are solely those of the authors and do not necessarily represent those of their affiliated organizations, or those of the publisher, the editors and the reviewers. Any product that may be evaluated in this article, or claim that may be made by its manufacturer, is not guaranteed or endorsed by the publisher.

## Footnotes

^1^ http://ftp.ncbi.nlm.nih.gov/genomes
