# Supplementary material for: Metagenomic-based pathogen surveillance for children with severe pneumonia in pediatric intensive care unit
Source: Front Public Health. 2023 Jun 15;11:1177069. doi: 10.3389/fpubh.2023.1177069 (PMC10309210; doi:10.3389/fpubh.2023.1177069)
Supplement: Supplementary file 1 [file Table_1.DOCX]

**Table S1 The potential pathogens co-detected with adenovirus**

|  | **Bacteria** | **Fungi** | **DNA viruses** | **RNA viruses** | **Others** |
| --- | --- | --- | --- | --- | --- |
| P2 |  | *Aspergillus fumigatus* | Adenovirus | Influenza A virus |  |
| P4 |  |  | Adenovirus |  |  |
| P5 |  |  | Adenovirus |  |  |
| P6 |  |  | Adenovirus |  |  |
| P11 |  |  | Adenovirus | Parainfluenza virus |  |
| P12 |  |  | Adenovirus | Influenza B virus |  |
| P13 |  |  | Adenovirus | Parainfluenza virus |  |
| P18 |  | *Candida albicans* | Adenovirus CMV | RSV |  |
| P20 | *Staphylococcus aureus* | *Candida albicans* | Adenovirus |  |  |
| P22 |  |  | Adenovirus |  | *Mycoplasma pneumoniae* |
| P24 |  |  | Adenovirus CMV | Parainfluenza virus |  |
| P25 |  |  | Adenovirus |  |  |
| P39 |  |  | Adenovirus |  |  |
| P42 | *Lactobacillus rhamnosus* |  | Adenovirus |  |  |
| P45 |  |  | Adenovirus |  |  |
| P48 |  |  | Adenovirus | RSV Human coronavirus OC43 |  |
| P77 |  |  | Adenovirus |  |  |
| P97 | *Klebsiella pneumoniae Enterococcus faecalis* |  | Adenovirus | RSV |  |
| P132 | *Haemophilus parainfluenzae* | *Candida albicans* | Adenovirus Human betaherpesvirus 7 CMV EBV |  |  |
| P206 | *Moraxella catarrhalis Haemophilus influenzae* |  | Adenovirus Torque teno mini virus | Coxsackievirus B3 |  |

**Table S2 The potential pathogens co-detected with *Mycoplasma pneumoniae***

|  | **Bacteria** | **Fungi** | **DNA viruses** | **RNA viruses** | **Mycoplasma** |
| --- | --- | --- | --- | --- | --- |
| P3 |  |  |  |  | *Mycoplasma pneumoniae* |
| P10 |  |  |  |  | *Mycoplasma pneumoniae* |
| P22 |  |  | Adenovirus |  | *Mycoplasma pneumoniae* |
| P41 |  |  | Human betaherpesvirus 6B |  | *Mycoplasma pneumoniae* |
| P52 |  | *Pneumocystis jirovecii* |  | RSV | *Mycoplasma pneumoniae* |
| P57 | *Streptococcus pneumoniae* |  |  |  | *Mycoplasma pneumoniae* |
| P232 | *Haemophilus influenzae* |  | Human betaherpesvirus 7 |  | *Mycoplasma pneumoniae* |
