# Supplementary figures and images for: Metagenomic-based pathogen surveillance for children with severe pneumonia in pediatric intensive care unit
Source: Front Public Health. 2023 Jun 15;11:1177069. doi: 10.3389/fpubh.2023.1177069 (PMC10309210; doi:10.3389/fpubh.2023.1177069)

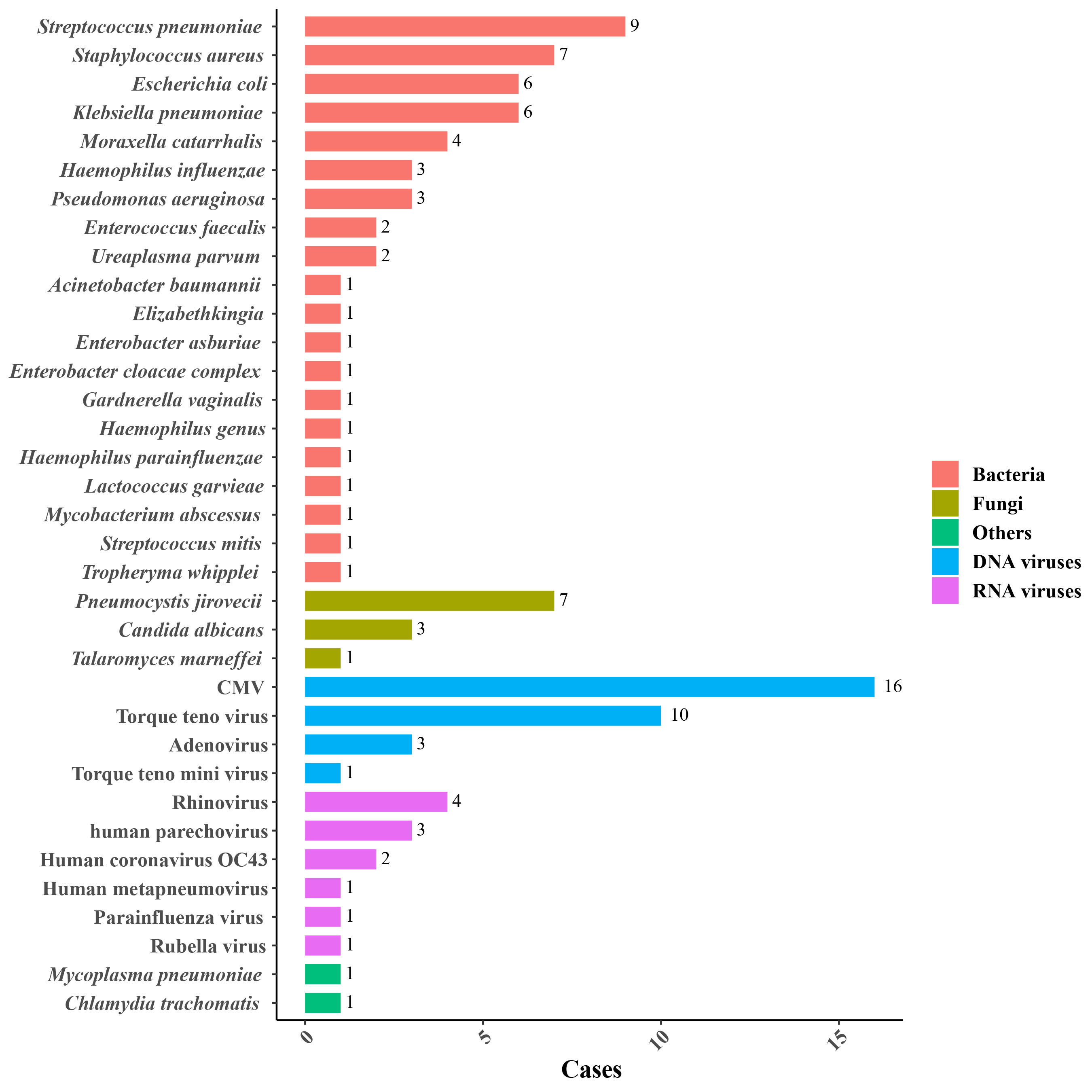

Supplement: Supplementary file 2 [file Image_1.JPEG]
